# Discovery of recurring slope lineae candidates in Mawrth Vallis, Mars

**DOI:** 10.1038/s41598-019-39599-z

**Published:** 2019-02-14

**Authors:** Anshuman Bhardwaj, Lydia Sam, F. Javier Martín-Torres, María-Paz Zorzano

**Affiliations:** 1 Division of Space Technology, Department of Computer Science, Electrical and Space Engineering, Luleå University of Technology, Luleå, Sweden; 2 Institut für Kartographie, Technische Universität Dresden, Dresden, Germany; 3 Instituto Andaluz de Ciencias de la Tierra (CSIC-UGR), Armilla, Granada, Spain; 4 UK Centre for Astrobiology, School of Physics and Astronomy, University of Edinburgh, Edinburgh, UK; 5 Centro de Astrobiología (INTA-CSIC), 28850 Torrejón de Ardoz Madrid, Spain

## Abstract

Several interpretations of recurring slope lineae (RSL) have related RSL to the potential presence of transient liquid water on Mars. Such probable signs of liquid water have implications for Mars exploration in terms of rover safety, planetary protection during rover operations, and the current habitability of the planet. Mawrth Vallis has always been a prime target to be considered for Mars rover missions due to its rich mineralogy. Most recently, Mawrth Vallis was one of the two final candidates selected by the European Space Agency as a landing site for the ExoMars 2020 mission. Therefore, all surface features and landforms in Mawrth Vallis that may be of special interest in terms of scientific goals, rover safety, and operations must be scrutinised to better assess it for future Mars missions. Here, we report on the initial detection of RSL candidates in two craters of Mawrth Vallis. The new sightings were made outside of established RSL regions and further prompt the inclusion of a new geographical region within the RSL candidate group. Our inferences on the RSL candidates are based on several morphological and geophysical evidences and analogies: (i) the dimensions of the RSL candidates are consistent with confirmed mid-latitude RSL; (ii) albedo and thermal inertia values are comparable to those of other mid-latitude RSL sites; and (iii) features are found in a summer season image and on the steep and warmest slopes. These results denote the plausible presence of transient liquid brines close to the previously proposed landing ellipse of the ExoMars rover, rendering this site particularly relevant to the search of life. Further investigations of Mawrth Vallis carried out at higher spatial and temporal resolutions are needed to identify more of such features at local scales to maximize the scientific return from the future Mars rovers, to prevent probable biological contamination during rover operations, to evade damage to rover components as brines can be highly corrosive, and to quantify the ability of the regolith at mid-latitudes to capture atmospheric water which is relevant for *in-situ*-resource utilization.

## Introduction

Mawrth Vallis has been a potential candidate landing site for several planned^1–3^ and previous Mars missions^4–7^. In particular until recently, Mawrth Vallis and Oxia Planum were the two final candidate sites selected for the European Space Agency’s (ESA’s) ExoMars 2020 rover mission before Oxia Planum was finally selected after robust scrutiny regarding surface and topographical conditions, and planetary protection implications. The terrain of Mawrth Vallis includes some of the oldest Martian rocks identified, and orbital observations confirm an abundance of phyllosilicates in the region, indicating that the rocks have developed from ancient wet clays in a neutral pH environment that could have favoured habitability^4,5^. Mawrth Vallis has been reported to exhibit several scientifically interesting advantages as a landing site^3^. The Mawrth Vallis region is extremely diverse in mineralogy^4,8^ and erosive lithology^4^ with ancient bedrocks and sediments^9,10^; it exhibits evidence of long lasting reducing conditions in regions of high clay content with biosignature preservation and habitability potential^5,11–13^; and the region is rich in phyllosilicates^14,15^ and has been reported to have sulfate precipitates^7,8^ in several locations, indicating the probable subsurface-surface fluidized flows and past surface water activity.

**Figure 1:**
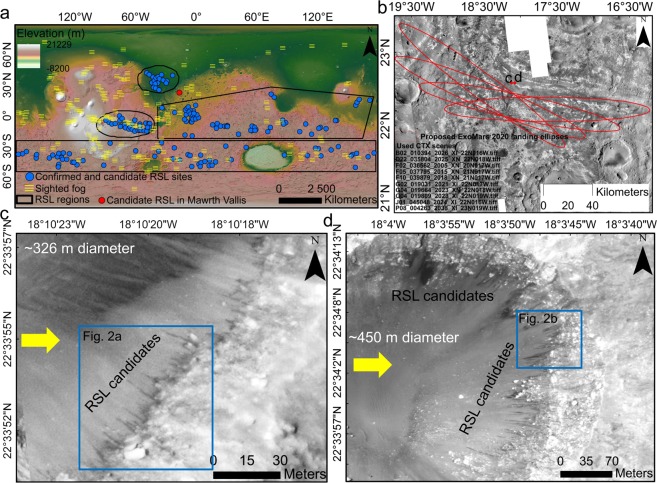
The locations of the RSL candidates. (**a**) Global distribution of sighted fog^35^, confirmed and candidate RSL sites^16,26,27,33,34^, and new candidate RSL sites in Mawrth Vallis. Mars Orbiter Laser Altimeter (MOLA) elevation and hillshaded view is in the background (courtesy: NASA/JPL/Goddard). (**b**) The locations of RSL candidates in the two craters (red circles mark contextual information for (**c**,**d**) within Mawrth Vallis with respect to proposed ExoMars 2020 landing ellipses (3-σ) and Context Imager (CTX) scenes for the region. (**c**,**d**) Craters on the left and the right, respectively, shown in HiRISE image ESP_045457_2025. Yellow arrows denote the direction of insolation against the RSL candidates (local Mars time: 15:17), thus proving them not to be topographic shadows. Blue rectangles provide contextual information for Fig. 2a,b. All maps are created using ArcGIS Version 10.4 (http://desktop.arcgis.com/en/arcmap/10.4/get-started/main/get-started-with-arcmap.htm). CTX image credit: NASA/JPL-Caltech. HiRISE image credit: NASA/JPL/University of Arizona.

Long after the 2012 landing of the Curiosity Rover in Gale Crater, Mars, several potential recurring slope lineae (RSL) candidates were reported on^16^, which spurred debate on plausible implications for rover surface operations. It seems that RSL may be more frequent and widespread than previously thought, though their small dimensions and the seasonal dependence of their appearance renders their orbital observation elusive. It is therefore desirable to make dedicated observations of such small-scale surface features at candidate rover landing sites, as their detection may facilitate certain scientific goals (especially if their presence is taken as an indicator of the plausible occurrence of transient liquid watery phases) and as such detection may also condition rover operations. In alignment with existing planetary protection regulations, our aim is to report on several candidates of RSL in Mawrth Vallis that have never been observed or reported on before. This discovery is particularly exciting because RSL have never been found in this entire region which has one of the thickest units of phyllosilicates and hydrated materials on Mars. RSL constitute a significant part of Martian special regions^17–19^. Additionally, there are conflicting opinions regarding whether RSL can be defined as watery flows^20–27^, dry events^28^, or a result of both^29,30^, as well as, on the probable amount of liquid water stored within them^21,29,31,32^. Our objective here is not to contribute to this discussion. The premise of the present work is to acknowledge the fact that RSL are considered within present planetary protection protocols and the possibility that they can be liquid water-related features, and thus here we: (i) report on new RSL candidate sites in Mawrth Vallis where RSL have never been observed before, (ii) offer evidence to examine the possibility of sighted RSL candidates qualifying as confirmed RSL sites, and (iii) discuss the probable implications of such detection for the future exploration of the Mawrth Vallis region. Independent of the potential implications for the future Mars missions, these new sightings carry a significance of their own as they are positioned outside of established RSL regions suggested by Stillman *et al*.^33^ (Fig. 1a), prompting the inclusion of a new geographical region of the RSL candidate group. This will have relevance for the future updating of existing RSL inventory with the continuous influx of more high-resolution images. A detailed discussion of RSL in relation to the observed candidates is given in the following sections.

## Results and Discussion

### Positional and morphological evidence for new RSL candidates

Figure 1b shows the relative locations of the two craters with respect to the proposed ExoMars 2020 landing ellipses and RSL candidate sites of Mawrth Vallis are observed on the HiRISE image ESP_045457_2025 (Fig. 1c,d). We have analysed the available 73 High Resolution Imaging Science Experiment (HiRISE) scenes until December 2017 (with latest acquisitions made in June 2016) for the region. Most of these scenes (~75% of all scenes) are onetime non-overlapping acquisitions for months that are not the hottest for this region. As is shown in Fig. 1a, the new RSL candidates are positioned ~1000 km away from the nearest confirmed RSL sites to the northwest and kilometres away from the nearest landing ellipse boundary (Fig. 1b). However, these sightings are relevant because they are made on the image acquired on the solar longitude (L_s_) 133.9° during the northern summer season unlike the majority of HiRISE acquisitions for this region. Only 20 of the analysed HiRISE scenes were acquired during different stages of the northern summer and most of them were taken during the late summer season when RSL fading has been reported from several of the confirmed RSL sites^33^. Thus, the RSL candidates identified suggest the possibility that more such observations for the Mawrth Vallis region and within the landing ellipse could be made with more scenes for the L_s_s that are most suitable for RSL initiation and growth. If we consider RSL as briny flows as suggested by past studies^20,23,25^, then this HiRISE image ESP_045457_2025 was captured in the midsummer season (7 April 2016; L_s_ 133.9°) when temperatures are neither too high to facilitate RSL fading nor too low to apply a constraint to its initiation. In addition, the fact that confirmed and candidate RSL sites (Fig. 1a) have been predominantly reported to the north and south of this region^16,26,27,33,34^ along with confirmed fog sightings (Fig. 1a) made nearby^35^ further illustrates the possibility that this region was undocumented for RSL mainly due to the unavailability of seasonally appropriate high resolution HiRISE images and not because RSL cannot form in Mawrth Vallis. At least three HiRISE observations for the same site made within two Martian years are needed to decide whether a candidate is a confirmed site^16^ and therefore we refer to our sightings as RSL candidates since we have only one HiRISE image available that covers the craters. Nevertheless, despite the unavailability of repeat images of these sites and in view of Mawrth Vallis being the favorite proposed landing site for the Mars missions, our singular observation deserves detailed investigation and prompt first-hand reporting.

**Figure 2:**
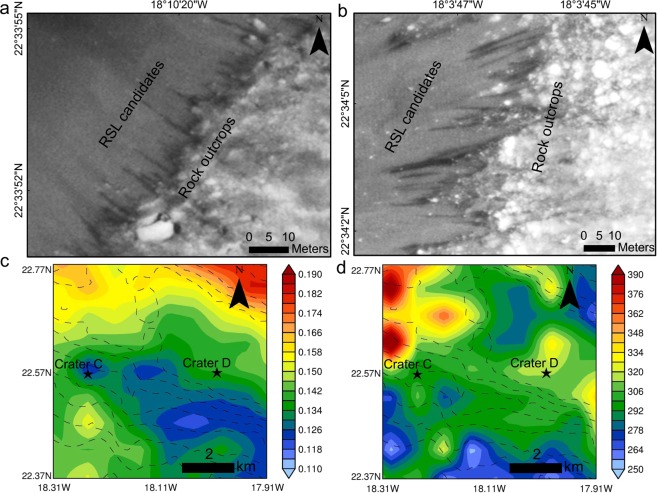
RSL candidates and rock outcrops shown in HiRISE image ESP_045457_2025 with contextual geophysical parameters derived from Thermal Emission Spectrometer (TES). (**a**) Crater C RSL candidates. (**b**) Crater D RSL candidates. Mars Global Surveyor (MGS) TES-derived albedo (**c**) and thermal inertia. (**d**) Contextual information for a and b can be derived from Fig. 1c,d, respectively. Dashed contours shown in (**c,d**) represent Mars Orbiter Laser Altimeter (MOLA) orography (negative) at 100 m interval. The unit of thermal inertia used in d is Jm^−2^K^−1^s^−1/2^. The maps were created using ArcGIS Version 10.4 (http://desktop.arcgis.com/en/arcmap/10.4/get-started/main/get-started-with-arcmap.htm). HiRISE image credit: NASA/JPL/University of Arizona.

The main criteria used to identify an RSL candidate based on published recommendations^16,23,34^ and RSL properties can be defined as follows: (i) ≥10 dark lineations resembling RSL (however, this threshold is arbitrary and is recommended to be dropped^16^), (ii) morphology and geologic settings of confirmed RSL, (iii) low albedo and high thermal inertia terrain, and (iv) observation made during warm seasons and on the warmest and steepest slopes. Image ESP_045457_2025 is not one of the highest resolution HiRISE images collected, as it has an original image scale range of 32.3 cm/pixel with 1 × 1 binning such that only objects of ~97 cm across are resolved. However, this resolution is sufficient to mark RSL candidates and to make estimates of their dimensions. The number of RSL candidates present in both craters exceeds the arbitrary threshold of 10 and signifies the possibility of mass slope activity. We found RSL candidates on the rims of two different craters at roughly the same latitudes (Fig. 1c,d) and ~6.5 km apart. To facilitate our discussion from here onwards, we refer to the crater shown in Fig. 1c as Crater C and to the crater shown in Fig. 1d as Crater D. Crater C is slightly smaller (~326 m) in diameter than Crater D (~450 m). We confirm that the observations made are not the result of the presence of any topographic shadows, as solar insolation is against the RSL candidate slopes at the time (local Mars time: 15:17) of image acquisition (Fig. 1c,d). We found all RSL candidates in craters on the warmest and steep slopes for the L_s_ of the acquired image and oriented towards western component (e.g., NW, W, and SW), reflecting the reported majority of RSL observed in Valles Marineris and at southern mid-latitudes^33^. In addition, all of the RSL candidates exhibit initiation below or adjacent to bedrock outcrops (Fig. 2), a typical marker of the majority of RSL^33^. The dimensions of these candidate RSL are consistent with those confirmed from other mid-latitude RSLs^27^ as their widths vary from ~0.5–3 m and as they can reach up to ~60 m in length. Mars Global Surveyor (MGS) Thermal Emission Spectrometer (TES)-derived albedo (Fig. 2c) values reveal that these craters have a low average albedo of only ~0.13, which is significantly lower than the regional average of ~0.2^36^ and which lies within the reported range^37^ of 0.1–0.17 (with a mean of 0.14) for the confirmed mid-latitude RSL sites. In addition, TES-derived thermal inertia (Fig. 2d) values for the craters show an average thermal inertia value of ~300 Jm^−2^K^−1^s^−1/2^, falling within the intermediate thermal inertia range of 150 to 460 Jm^−2^K^−1^s^−1/2^ recorded for much of this region^36^, and consistent with the range^21^ of 250–450 Jm^−2^K^−1^s^−1/2^ reported for RSL sites with sandy slopes and small outcrops or boulders as shown for Craters C and D and with the average 240 Jm^−2^K^−1^s^−1/2^ value reported^37^ for mid-latitude RSL sites. Here, it is worth mentioning that albedo and thermal inertia vary locally in this region as the geologic units are of different origins and consist of different surface materials such as boulders at the top of the crater flank, dust on the slopes, and coarser sand on the crater floor. However, a recent study^38^ based on Mars Science Laboratory (MSL) Rover Environmental Monitoring Station (REMS) data simulations has shown that even geological units of thermal inertia less than 185 Jm^−2^K^−1^s^−1/2^ for a broad range of albedo values can form surface brines while at less than 175 Jm^−2^K^−1^s^−1/2^ of thermal inertia and a relatively high albedo of 0.25, they can occasionally favour subsurface brine production through deliquescence. This conclusion is relevant in view of proposed hypotheses of deliquescence-based brine formation for RSL initiation^20–25^.

**Figure 3:**
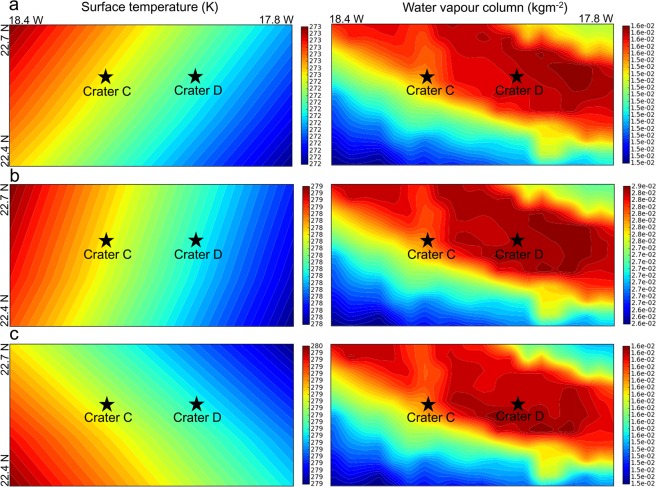
Mars Climate Database (MCD) V5.3 simulations^39,40^ of surface temperature and water vapour columns for local Mars time 15:00. (**a**) L_s_ 90°. (**b**), L_s_ 135°. (**c**) L_s_ 180°. MCD v5.3 can be accessed from, http://www-mars.lmd.jussieu.fr. MCD credits: LMD/OU/IAA/ESA/CNES.

Further consideration of these wet deliquescence-based hypotheses on RSL formation^20–25^ in the observed craters requires making estimates of two main atmospheric factors, i.e., temperature and atmospheric water abundance, which govern such deliquescence-based processes. Figure 3 clearly reveals that if RSL are deliquescence-based briny features then the areas in which they have been observed in Mawrth Vallis and the time (L_s_) of observations are the most appropriate. We used Mars Climate Database (MCD) V5.3 (http://www-mars.lmd.jussieu.fr) simulations^39,40^ of surface temperature and water vapour column for local Mars time 15:00 (the time of HiRISE image ESP_045457_2025 acquisition) and for L_s_ 90° (start of the northern summer), L_s_ 135° (middle of the northern summer; also close to the L_s_ of HiRISE image ESP_045457_2025), and Ls 180° (end of the northern summer). Afternoon surface temperature conditions for both craters are moderate throughout the summer and in agreement with the reported range of 250–300K for mid-latitudes^41^ and should not constrain deliquescence. However, during the middle of the summer (Fig. 3b) and even in the afternoon water vapour column values are nearly twice those measured at the start (Fig. 3a) and end of the summer (Fig. 3c). This significant midseason increase in water vapour does not only imply a higher probability of surficial or sub-surficial deliquescence but it also indicates the potential presence of a local atmosphere-regolith interaction taking the form of an active water cycle. However, to comment on these possibilities with higher confidence, better spatiotemporal resolution simulations for relative humidity using suitable water vapour mixing ratios and even higher resolution topography are needed, though this is not our present objective. Instead, with Fig. 3 we intend to establish that the sighted candidates are present in a region in which deliquescence possibilities are highest within Mawrth Vallis and that such features are observable in HiRISE image ESP_045457_2025 because this image was captured during the most appropriate time of the summer in this region with favourable temperatures and water vapour abundance in the atmosphere for potential brine formations^42^.

### Possible surficial trends at RSL candidate sites

Before starting this section, we emphasise that the present work is primarily about reporting RSL candidates in a new region with one of the thickest units of phyllosilicates and hydrated materials on Mars^43^ and a rich aqueous history^44–49^. Now, the conditions to call these dark albedo features a RSL “candidate” as proposed by the published literature^16,23,34^ have already been fulfilled through the discussions on positional and morphological characteristics in the previous section. Thus, the present section discussing possible surface characteristics at and around RSL candidate sites should be regarded as additional discussion for interested readers and is prompted more as a future scope. All previously reported RSL candidate features have been identified as such based only on their geomorphology, location, and seasonal state, and thus the same criteria have been applied here. For this preliminary surficial investigation of the RSL candidate slopes, we were fortunate that the only Mars Reconnaissance Orbiter (MRO) Compact Reconnaissance Imaging Spectrometer for Mars (CRISM) image (product id is FRT00003BFB_07_BRFALJ_MTR3) for this part of Mawrth Vallis was acquired during the northern summer (Ls 161.543°) (5 January 2007) and that it completely covered Crater C. We used thematically related summary products for these data as red-green-blue (RGB) colour composites called Browse Products that allow for the fast visual and qualitative multi-parametric assessment of surface characteristics and spectral variability using the information content of the calibrated image cube^50,51^. These summary products are georeferenced, offer the best hyperspectral spatial resolution of 18 m/pixel for the Martian surface, and although they are carefully developed using composites of most relevant spectral bands to avoid false positives from spectrally similar minerals, we suggest to opt for a cautious approach while interpreting them in the present case. These datasets can be rendered and visualised on JMARS software^52^. The best possible way to confirm these visual interpretations would have been to perform detailed spectral analyses by matching the spectra with signatures in available spectral library (http://crismtypespectra.rsl.wustl.edu/). However, this detailed mineralogical spectral analyses can be subject of a separate paper altogether and is beyond the scope of the present work. Moreover, published literature already provides significant overview of the mineralogical characteristics of the Mawrth Vallis region^44–49,53^. The Mawrth Vallis region shows large outcrops of light-toned sedimentary layered deposits^44,45,53^ with near-infrared spectral characteristics which are consistent with a variety of hydrated minerals associated with these layered rocks^53^. In fact, Observatoire pour la Minéralogie, l’Eau, les Glaces et l’Activité (OMEGA) spectra modelling has revealed a large fraction of clay minerals in these layered outcrops with clay mineral abundances for several outcrops reaching as high as half of the entire mineral composition^53^. These layers display a typical compositional stratigraphy in which Fe/Mg smectites are overlaid by Al-smectites^15^, followed by kaolinite and hydrated silica^14^. As a separate mineralogical entity, sulfates have also been reported locally for Mawrth Vallis^8^.

**Figure 4:**
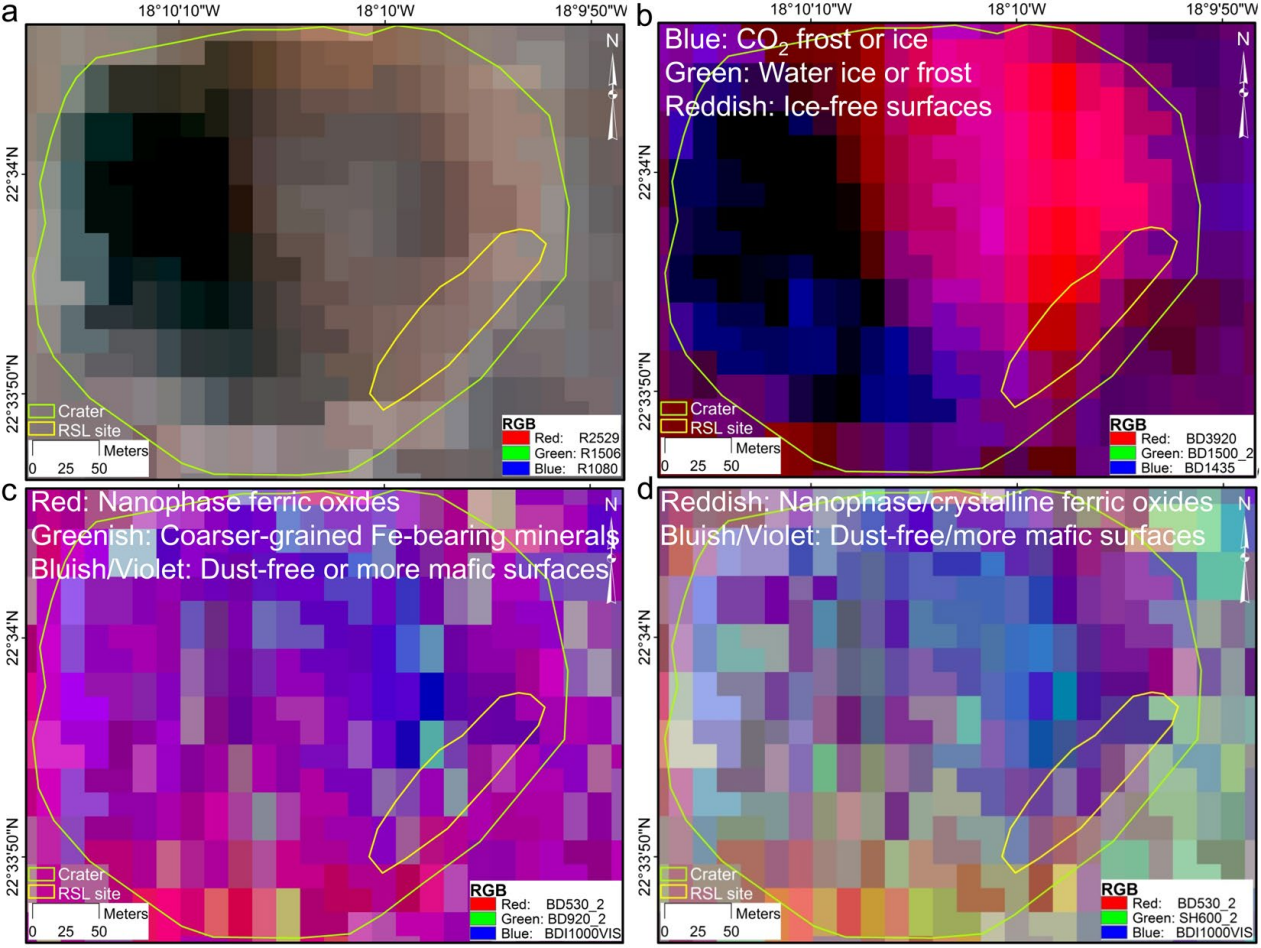
Mars Reconnaissance Orbiter (MRO) Compact Reconnaissance Imaging Spectrometer for Mars (CRISM) Map Projected Targeted Reduced Data Record (MTRDR) Browse products^51^ for Crater C highlighting ice-free and dust-free conditions. (**a**) An enhanced infrared false colour representation of the scene for contextual information. (**b**–**d**) show the ice-free and dust-free nature of the slopes. (**b**) is derived from the Version 2 Ices product, (**c**) is derived from the Version 2 Fe Mineralogy product, and d is derived from the Fe Mineralogy product. The CRISM product id is FRT00003BFB_07_BRFALJ_MTR3, the image was acquired on L_s_ 161.542883° (5 January 2007), and the products used can be downloaded from, http://ode.rsl.wustl.edu/mars/indexproductpage.aspx?product_id=FRT00003BFB_07_IF166J_MTR3&product_idGeo=25111991. The maps were created using ArcGIS Version 10.4, (http://desktop.arcgis.com/en/arcmap/10.4/get-started/main/get-started-with-arcmap.htm). CRISM image credit: NASA/JHUAPL/JPL/University of Arizona.

Here, through these Browse Products, we are only aiming to provide first-hand surficial trends for the craters where RSL candidates have been sighted. While even 18 m/pixel resolution of these Browse Products is not sufficient for investigating the RSL candidates individually, we focused on investigating the entire slope (blue rectangle in Fig. 1c) on which RSL candidates were observed (Fig. 4, Supplementary Figs 1 and 2) with the assumption that these RSL candidates originate on the same slopes in the northern summers of every Martian year. While Browse Products might not be useful for definite and quantitative mineral assessments, for preliminary qualitative analyses they are useful as the depth of an absorption feature associated with a specific mineral typically scales with its local abundance^51^. Legend information given on colours and associated mineralogy (Fig. 4, Supplementary Figs 1 and 2) is directly derived from the MTRDR label file included with each of the Browse Products. Here, we were interested in performing quick qualitative analyses of the surface potentially present in the regolith of the crater slopes, and in validating our analyses, as previously suggested^51^, we used a combination of various Browse Products for the same slopes to observe mineralogical agreement between them. The confirmed RSL sites show a relatively dust-free or low-dust lithology^33,37^. Now two of the Browse Products (Fe Mineralogy and Version 2 Fe Mineralogy) directly provide information on dust conditions and as we see in Fig. 4c,d, the RSL candidate slopes are largely dust-free. We further confirm and quantify this using TES-derived dust cover index (DCI) which suggests a mean DCI value of 0.97, consistent with confirmed RSL sites found at mid-latitudes^37^. For a local Mars time of ~15:00, these slopes are largely ice- or frost-free as determined from the Version 2 Ices product (Fig. 4b). The absence of detected water ice or frost could be explained by a completely dry surface or with water (liquid or frozen) amount that is below the detection limits.

### Conclusions and implications for future exploration

The RSL candidates found in Mawrth Vallis and the evidence provided here linking them to the confirmed RSL can have profound implications for the future rover-based exploration of the region. More research is needed to understand the local-scale abundance of chlorides or perchlorates^54–56^ on Mars and their significance for liquid water-based hypotheses explaining RSL formation or its propagation. Furthermore, the capacities for such liquid brines to support lifeforms on Mars^29,57,58^ is also debatable. However, under the current planetary protection policies, RSL hold a significant place^19^ in the delimitation of the so-called Special Regions of Mars. Due to its considerably variable and rich mineralogy, Mawrth Vallis has always been a favourite site for rover-based missions. While such sightings of RSL candidates can place some constraints on deciding the rover traverses following planetary protection protocols, they can equally excite science teams involved with rover instruments^59^, as these RSL candidates signify a possible local-scale active water cycle and regolith-atmosphere interactions. Our results are relevant in providing an initial reporting of such RSL candidates from the Mawrth Vallis region and prompt more detailed and focused investigations in this regard. We found a considerable lack of seasonal and temporal HiRISE and CRISM coverage for the same areas of Mawrth Vallis, and our results can stimulate the involved research teams of these instruments in engaging in more acquisitions in the near future to further clarify the relevance of present observations.

If RSL are a result of briny flows as suggested by past studies, then such brines (particularly Cl-bearing brines) can be extremely corrosive and can significantly damage rover components. Such probable processes of corrosion in the Martian conditions are some of the least studied, and the development of corrosion resistant materials for rover manufacturing must account for the fact that corrosion processes or rates observed on Mars can be different from those observed on Earth^60^. It would be of particular interest to implement further close range investigations of RSL-like slope processes using such corrosion resistant rovers and equipment, to provide ground-truth to orbital investigations and to then extend our understanding of what may be occurring at other RSL sites on Mars. Irrespective of the fact that Mawrth Vallis was not chosen as the final landing site for the ExoMars 2020 mission, these new sightings carry a significance of their own, as they were made outside of the established RSL regions^33^ (Fig. 1a) and prompt the inclusion of a new geographical region in the RSL candidate group. In future mars missions, most landings will take place at low or mid-latitudes because high latitudes offer little solar energy throughout operation time. Thus, wide-scale “RSL hunting” is crucial to identify such regions of interest, which exhibit potential for enhanced water capture and release and which may act as local water resources and preferable sites for sampling on Mars for future Mars sample return missions^61^. Furthermore, the existence of features where atmospheric water absorption might be taking place at present day conditions, is of interest for the future sustained exploration of Mars, as these sites may allow for implementing water harvesting strategies and water is one of the critical products required from *in-situ* resource utilization^62–64^. Finally, in view of the ESA and the National Aeronautics and Space Administration’s (NASA’s) recent declaration of interest in cooperating on the Mars sample return programme^61^, many lessons may be learned from the planning and execution of the two next rover missions that will be operating on Mars simultaneously: the Mars-2020 NASA rover and the ExoMars 2020 rover of the ESA. In particular, these rovers shall provide ground truth to orbital measurements on the wet/dry state of the soil and on the plausible habitability of samples that may be at reach of drillers and arms.

## Materials and Methods

The methodology adopted for the present study was straightforward and simple. We examined available HiRISE images of Mawrth Vallis to identify landforms that provide information on past and present hydrological conditions observed in the region. Serendipitously, in HiRISE scene ESP_045457_2025 we observed dark narrow streaks on the slopes of two craters and we initiated this study with the premise that these streaks were candidate RSL. We performed the dimensional measurements of these RSL candidates using ArcGIS Version 10.4 (http://desktop.arcgis.com/en/arcmap/10.4/get-started/main/get-started-with-arcmap.htm) software. Albedo and thermal inertia estimates made for the region were derived from TES data available from the Planetary Data System (http://pds.nasa.gov). Temperature and water vapour abundance simulations were carried out using the online version of Mars Climate Database (MCD) V5.3 (http://www-mars.lmd.jussieu.fr). CRISM Browse Products used in this study can be directly downloaded at http://ode.rsl.wustl.edu/mars/indexproductpage.aspx?product_id=FRT00003BFB_07_IF166J_MTR3&product_idGeo=25111991. Colour coding systems used to decipher mineralogy can be found within respective MTRDR label file for each CRISM Browse Product.

### Ethical approval and informed consent

We confirm that this is a completely remote sensing-based interplanetary study and does not involve any biological experiments.

## Supplementary information

Supplementary Information

## Data Availability

All of the remote sensing data used in this study are freely available from the Planetary Data System (http://pds.nasa.gov) and from sensor-specific websites such as http://hirise.lpl.arizona.edu/ and http://crism-map.jhuapl.edu/. All the data products analysed for this study are included as web references in the figure captions and text of this article.
