# Supplementary material for: Discovery of recurring slope lineae candidates in Mawrth Vallis, Mars
Source: Sci Rep. 2019 Feb 14;9:2040. doi: 10.1038/s41598-019-39599-z (PMC6376049; doi:10.1038/s41598-019-39599-z)
Supplement: Supplementary file 1 — Supplementary Information [file 41598_2019_39599_MOESM1_ESM.pdf]

Supplementary information

Discovery of recurring slope lineae candidates in Mawrth Vallis, Mars

Anshuman Bhardwaj[1*], Lydia Sam[1,2], F. Javier Martín-Torres[1,3,4], María-Paz Zorzano[5,1]

[1]Division of Space Technology, Department of Computer Science, Electrical and Space Engineering, Luleå University of Technology, Luleå, Sweden
[2]Institut für Kartographie, Technische Universität Dresden, Germany
[3]Instituto Andaluz de Ciencias de la Tierra (CSIC-UGR), Armilla, Granada, Spain
[4]UK Centre for Astrobiology, School of Physics and Astronomy, University of Edinburgh, UK
[5]Centro de Astrobiología (INTA-CSIC), 28850 Torrejón de Ardoz, Madrid, Spain
[*]Corresponding Author (anshuman.bhardwaj@ltu.se)

In this supplementary information, we discuss several preliminary mineralogical inferences based on the CRISM Browse Products and as we have mentioned in the main text, these inferences should be accepted with caution. Detailed spectral analyses are required to further confirm these preliminary visual observations. After observing a possible dust- and ice-free nature of the slopes using various Browse Products, we further focused on inferring potential abundant mineralogical and ionic species on the RSL candidate slopes. Our knowledge about a defined or common mineralogy for all the confirmed RSL sites is presently very limited and the reported mineralogy varies widely. Therefore, deriving any mineralogical congruence with confirmed RSL sites is not our objective here but we aim to provide a first-hand information on mineralogical possibilities at the RSL candidate sites to highlight the presence of possible cationic or anionic species which can form strong eutectic salts, capable of causing enough deliquescence to generate RSL, if RSL are briny features. Supplementary Fig. 1 presents a combination of several Browse Products for Crater C. The Mafic Mineralogy product (Supplementary Fig. 1a) suggests the possibly high presence of olivine and/or Fe-phyllosilicates. Olivine has not been reported in this region using Observatoire pour la Minéralogie, l'Eau, les Glaces et l'Activité (OMEGA) data probably due to higher detection limits. As noted above, the entire Mawrth Vallis region is rich in phyllosilicates, but their association with Fe shows an abundance of Fe as well, and such phyllosilicates have also been reported from RSL-abundant slopes and from within the Asimov Crater[34]. This also highlights the possible presence of high regolith hydration levels as these are sheet silicates that in addition to chlorite, micas and serpentine can also incorporate other clay minerals. Similarly, a potentially high presence of olivine in the regolith further points toward an abundance of Mg and Fe, as olivine is primarily Mg-Fe silicate. The solid-solution series of olivine most commonly consists of Mg-rich forsterite (Mg_2SiO_4) and Fe-rich fayalite (Fe_2SiO_4)[S1] and the relative abundance of Mg/Fe content within this series is denoted by its forsterite number (Fo #)[S1]. Fo # is defined[S1] as molar Mg/(Mg+Fe) X 100. To further corroborate the olivine abundance as shown in the CRISM Mafic Mineralogy product, we rendered TES-derived olivine map[S1] based on Fourier transform Michelson interferometry (Supplementary Fig. 3) for the craters as well as the surroundings in JMARS[52] software and this analysis highlights that (1) olivine within Mawrth Vallis has one of the highest concentrations (~8%) in and around the reported craters, and (2) the Fo # of 42-57 for the observed olivine signifies that it is equally rich in both Fe and Mg. The possibly high availability of Mg and Fe cations in the regolith is further recognised by Fe and Mg Phyllosilicate product (Supplementary Fig. 1b) revealing a significant presence of Mg/Fe-smectites (another class of phyllosilicates) and Mg carbonate in the regolith. As a final cross-check, we examined the Carbonate product (Supplementary Fig. 1c) and again observed profound levels of Fe and Mg Phyllosilicates. The significance of the presence of chlorides as an anionic species that can form salts ideal for brine formation has been well-studied[42,54,55]. The confirmed presence of chlorides on the reported RSL candidate slopes cannot be made using CRISM Browse Products alone. A thorough spectral analysis of the near-infrared (NIR) spectrum of the region should be made. However, here we take a look at the Inferred Chloride Deposits product (Supplementary Fig. 1d) and observe that on crater slopes with candidate RSL (predominantly west facing), chloride salts and related mineral deposits might be prevalent. A previous analysis[24] based on

2001 Mars Odyssey Thermal Emission Imaging System (THEMIS) data, with a spatial resolution of ∼100 m/pixel, did not spectrally detect chloride salts at RSL sites probably due to data resolution limitations. As we see in the present case, chlorides are locally present on west (NW, W, and SW) facing slopes only and Crater C can entirely get covered within 3 - 4 THEMIS pixels and thus can never provide a positional or qualitative presence of chlorides. Nevertheless, here our main focus is to observe if there is possibly higher overall abundance of regolith Cl in the regions where our RSL candidate craters are present and we performed an additional observation on data from the Gamma Ray Spectrometer (GRS) aboard the 2001 Mars Odyssey (https://grs.lpl.arizona.edu/grs-web/specials/Smoothed_rebinned_map_data)[56]. Supplementary Fig. 4 shows Cl-abundance in Mawrth Vallis as weight% (wt%) value. The highest wt% values in lower and middle latitudes on Mars[54] (±60°) vary between 0.2 – 0.8% and we observe that Mawrth Vallis region is relatively abundant in Cl. However, the Cl wt% in the region where RSL candidate craters are present is in the top quartile of the wt% values (0.51%), thus displaying relatively higher Cl-abundance in the regolith. From these observations, we can infer that possibly prevalent cationic species found in and around the study site include Fe^{2+}/Fe^{3+} and Mg^{2+} while a possible anionic dominance of Cl^- cannot be ruled out, although it will need further detailed spectral investigations. Possibilities of such occurrences in contexts of RSL have been discussed previously[20,23,25,27].

We further searched for possible hydrated minerals on the candidate slopes. In the past, such hydrated minerals have been found on several of the confirmed RSL sites during RSL activity[25] via temporal hyperspectral observations. Here, we have only one CRISM image available, and thus we cannot with certainty comment on the association of hydrated minerals with RSL activity to further derive brine plausibility. However, using this CRISM mineralogical information for RSL candidate slopes, we can observe a possibly high abundance of hydrated minerals (Supplementary Fig. 2). The Hydrated Mineralogy product (Supplementary Fig. 2a) reveals the possible acceptable levels of hydrated minerals with traces of monohydrated sulfates. The Hydrated Silica product (Supplementary Fig. 2b) additionally shows possibility of Al-OH minerals and of other hydrated minerals on these slopes. The Ices product (Supplementary Fig. 2c) further corroborates it showing possible presence of acceptable levels of hydrated minerals. To additionally corroborate the possibility of hydrated minerals, we also used the Phyllosilicates product (Supplementary Fig. 2d) to find that most pixels within the candidate slope boundary were identified as magenta, brown, and green, colours denoting the possible presence of hydrated Mg/Fe-OH minerals and Al/Si-OH-bearing minerals. Thus, while these observations support each other for a possible abundance of Fe and Mg minerals present on these slopes as highlighted in Supplementary Fig. 1, they also imply the possible presence of high hydration levels within these minerals during the season in which RSL candidates have been observed. However, as mentioned in the beginning of this mineralogical analysis, presently it is highly qualitative as the CRISM Browse Products do not document mineral identifications with a certainty, but they primarily indicate trends in the spectral parameters. While these spectral trends are presently interesting and corroborating the previous reporting on confirmed RSL sites, we need detailed spectral analyses as a future scope of the present work to confirm the mineralogy at the study sites.

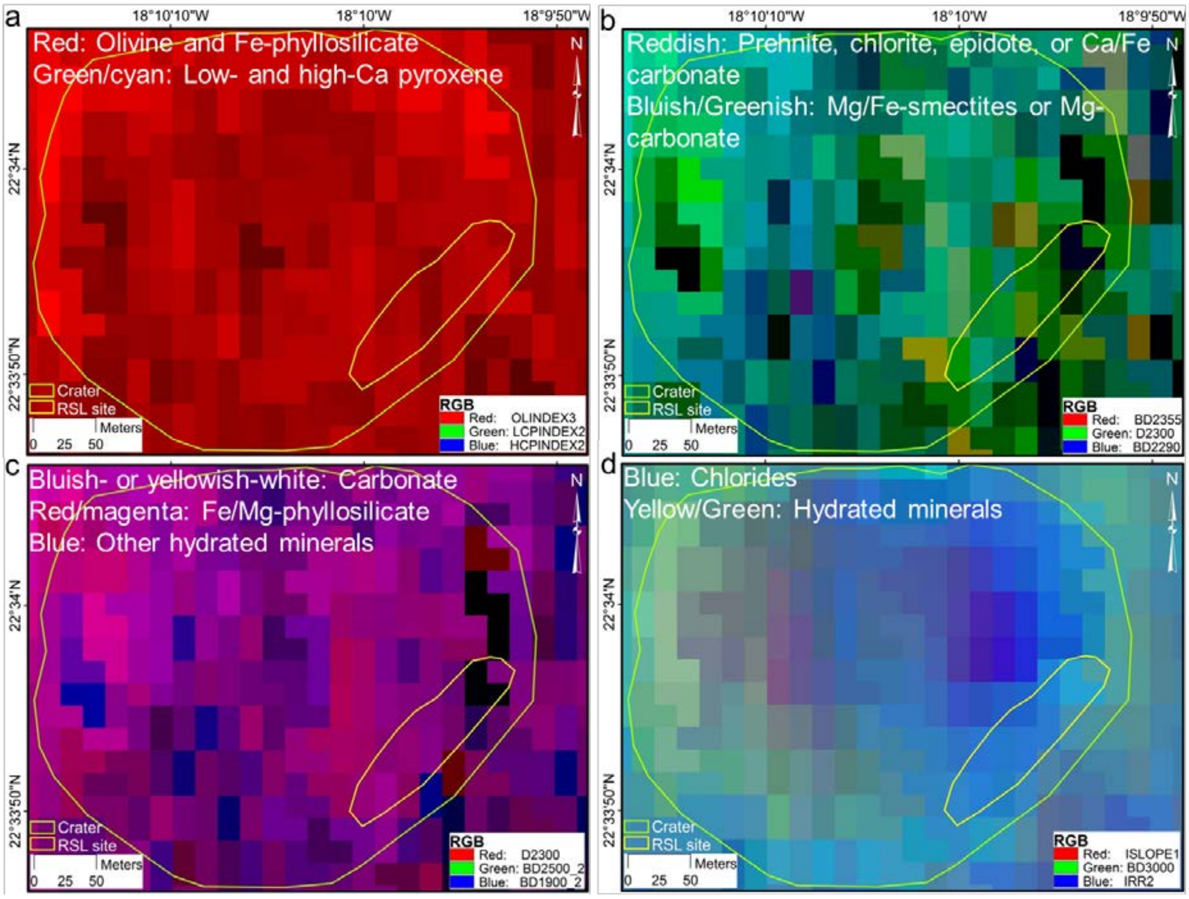

Supplementary Fig. 1 | MRO CRISM MTRDR Browse products[51] for Crater C highlighting Mg, Fe, and Cl rich mineralogy on the RSL candidate slopes. a, Mafic Mineralogy product. **b,** Fe and Mg Phyllosilicate product. **c,** Carbonate product. **d,** Inferred Chloride Deposits product. The CRISM product id is FRT00003BFB_07_BRFALJ_MTR3, the image was acquired at L_s 161.542883° (5 January 2007) and the products used can be downloaded from http://ode.rsl.wustl.edu/mars/indexproductpage.aspx?product_id=FRT00003BFB_07_IF166J_MTR3&product_idGeo=25111991. The maps were created using ArcGIS Version 10.4 (http://desktop.arcgis.com/en/arcmap/10.4/get-started/main/get-started-with-arcmap.htm). CRISM image credit: NASA/ JHUAPL/JPL/University of Arizona.

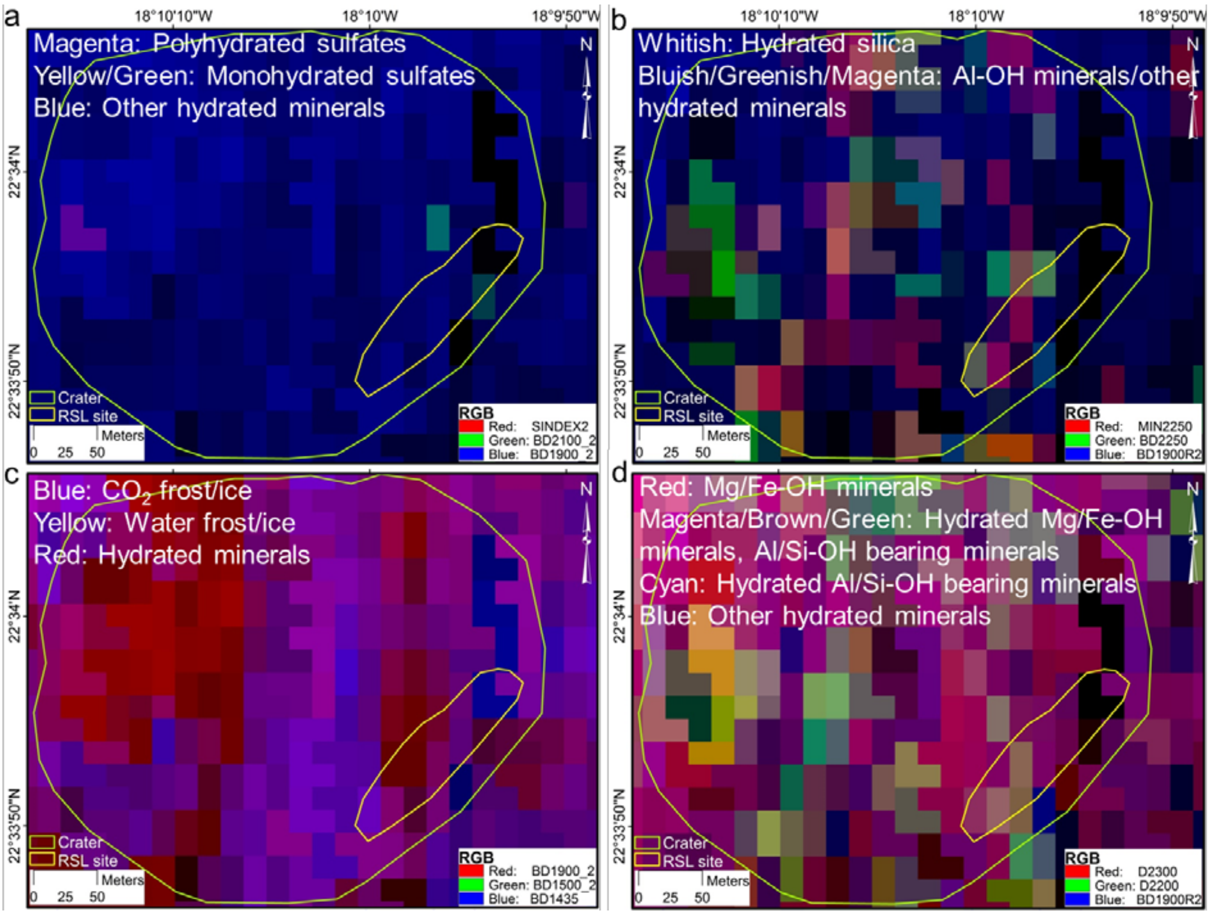

Supplementary Fig. 2 | MRO CRISM MTRDR Browse products[51] for Crater C highlighting an abundance of hydrated minerals found on the candidate RSL slopes. a, Hydrated Mineralogy product. **b,** Hydrated Silica product. **c,** Ices product. **d,** Phyllosilicates product. The CRISM product id is FRT00003BFB_07_BRFALJ_MTR3, the image was acquired at L_s 161.542883° (5 January 2007) and the products used can be downloaded from http://ode.rsl.wustl.edu/mars/indexproductpage.aspx?product_id=FRT00003BFB_07_IF166J_MTR3&product_idGeo=25111991. The maps were created using ArcGIS Version 10.4 (http://desktop.arcgis.com/en/arcmap/10.4/get-started/main/get-started-with-arcmap.htm). CRISM image credit: NASA/ JHUAPL/JPL/University of Arizona.

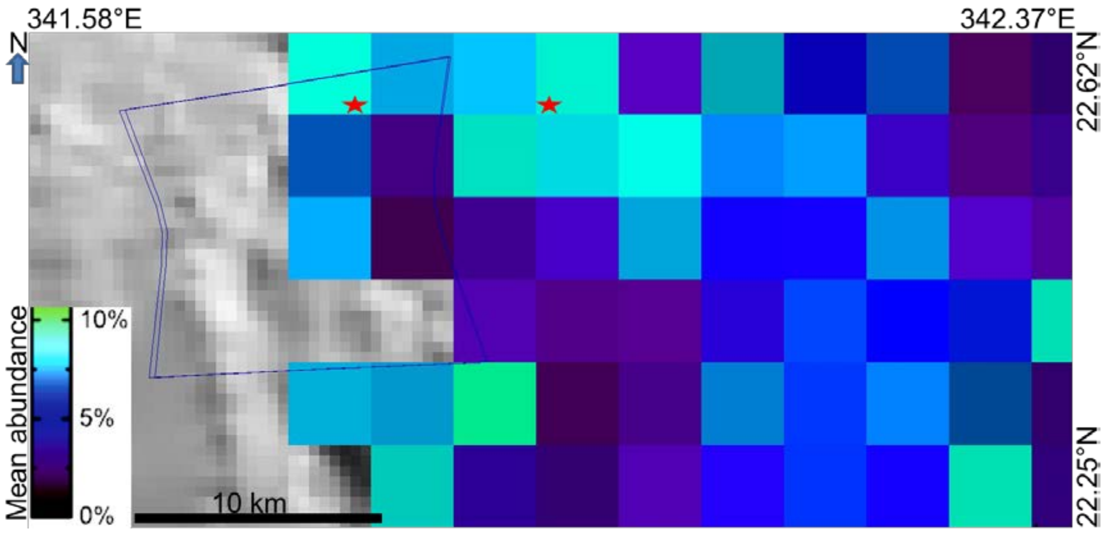

Supplementary Fig. 3 | Surface olivine abundance index numerical map for forsterite numbers 42 to 57 (Fo42 to Fo57) as derived from selected TES spectra[S1]. The blue polygon is the outline stamp of CRISM FRT00003BFB scene and the red stars show the locations of the two craters with candidate RSL. Mars Orbiter Laser Altimeter (MOLA) elevation data is in the background. This data is rendered and exported as an image from JMARS (https://jmars.asu.edu/) freeware[52]. JMARS image credit: NASA/JPL-Caltech/Arizona State University.

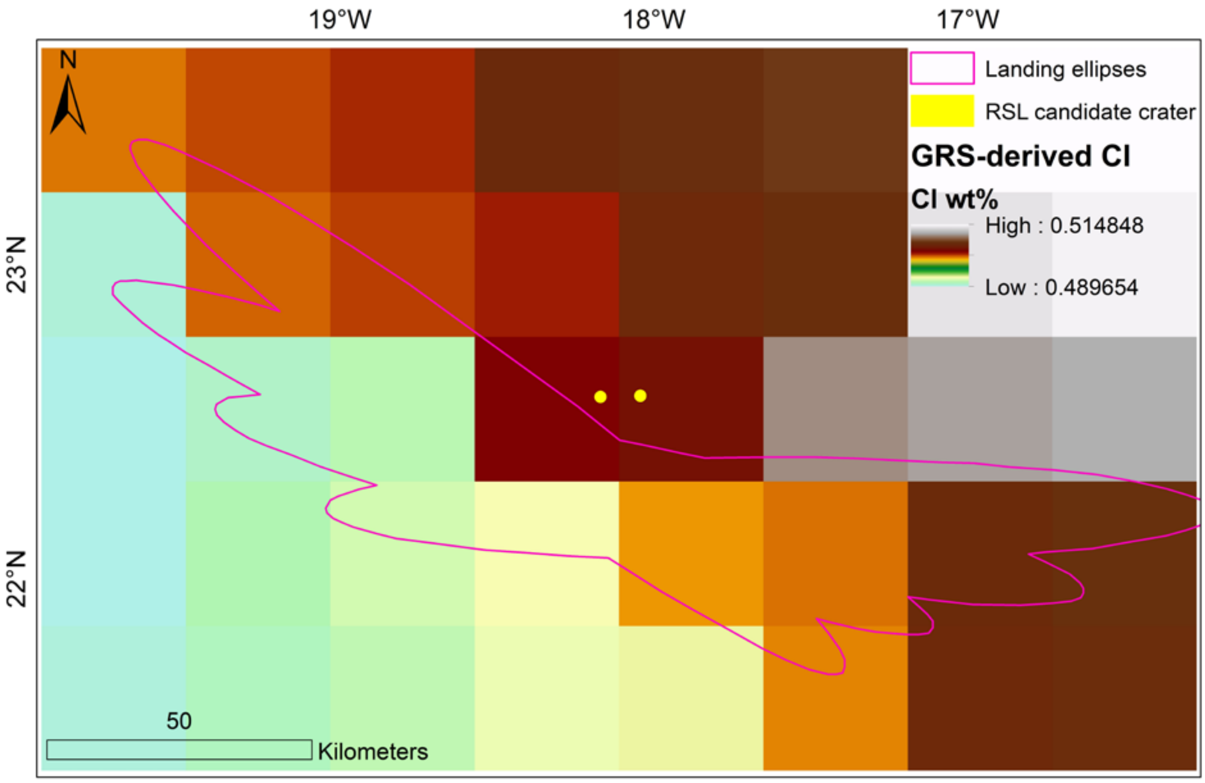

Supplementary Fig. 4 | Cl-abundance in Mawrth Vallis. The Cl weight% data was obtained from https://grs.lpl.arizona.edu/grs-web/specials/Smoothed_rebinned_map_data. The map was created using ArcGIS Version 10.4 (http://desktop.arcgis.com/en/arcmap/10.4/get-started/main/get-started-with-arcmap.htm).

Reference

S1. Koeppen, W. C. & Hamilton, V. E. Global distribution, composition, and abundance of olivine on the surface of Mars from thermal infrared data. *Journal of Geophysical Research: Planets*, **113(05001)** (2008).
